# Transcranial Current Stimulation of the Temporoparietal Junction Improves Lie Detection

**DOI:** 10.1016/j.cub.2015.08.014

**Published:** 2015-09-21

**Authors:** Sophie Sowden, Gordon R.T. Wright, Michael J. Banissy, Caroline Catmur, Geoffrey Bird

**Affiliations:** 1 MRC Social, Genetic, and Developmental Psychiatry Centre, Institute of Psychiatry, Psychology, and Neuroscience, King’s College London, London SE5 8AF, UK; 2 Department of Psychological Sciences, Birkbeck, University of London, London WC1E 7HX, UK; 3 Department of Psychology, Goldsmiths, University of London, London SE14 6NW, UK; 4 Institute of Cognitive Neuroscience, University College London, London WC1N 3AR, UK; 5 School of Psychology, University of Surrey, Guildford GU2 7XH, UK

## Abstract

The ability to detect deception is of vital importance in human society, playing a crucial role in communication, cooperation, and trade between societies, businesses, and individuals. However, numerous studies have shown, remarkably consistently, that we are only slightly above chance when it comes to detecting deception [1]. Here we investigate whether inconsistency between one’s own opinion and the stated opinion of another impairs judgment of the veracity of that statement, in the same way that one’s own mental, affective, and action states, when inconsistent, can interfere with representation of those states in another [2]. Within the context of lie detection, individuals may be less accurate when judging the veracity of another’s opinion when it is inconsistent with their own opinion. Here we present a video-mediated lie-detection task to confirm this prediction: individuals correctly identified truths or lies less often when the other’s expressed opinion was inconsistent with their own (experiment 1). Transcranial direct current stimulation (tDCS) of the temporoparietal junction (TPJ) has previously been shown to improve the ability to selectively represent the self or another [3–5]. We therefore predicted that TPJ stimulation would enable lie detectors to inhibit their own views, enhance those of the other, and improve their ability to determine whether another was presenting their true opinion. Experiment 2 confirmed this second prediction: anodal tDCS of the TPJ improved lie detection specifically when one’s own and others’ views were conflicting.

## Highlights

• Inconsistency between one’s own and another’s stated opinion impairs lie detection
• Stimulation of the right TPJ improves lie detection in opinion-inconsistent situations

## Teaser

Sowden et al. demonstrate how a mechanism involved in inhibiting imitation and in perspective taking extends to lie detection. They show that inconsistency between the opinions of the self and another impedes lie detection and that electrical stimulation of the temporoparietal junction improves performance in these opinion-inconsistent situations.

## Results and Discussion

Despite the frequency of deception in everyday life [6] and the importance of detecting deception within human society, humans are remarkably consistent in their inability to detect deception. Meta-analyses demonstrate a mean success rate of 54% across all published studies of lie detection ability, where chance performance is 50%, with a measurement-corrected SD of just 0.8% [1]. Although one or two cues have been demonstrated to signal deception at above-chance levels in some studies (e.g., response latency [7]), these cues are mostly overlooked in favor of non-diagnostic behavioral cues (such as the avoidance of eye contact [8]) or person-level cues relating to the perceived deceptiveness of the individual rather than what they are saying (“demeanor bias” [9]), resulting in poor performance. Here we investigate the existence of a further factor that may decrease the accuracy of lie detection when one is attempting to determine the veracity of another’s stated opinion: inconsistency between one’s own opinion and that of another.

**Figure 1:**
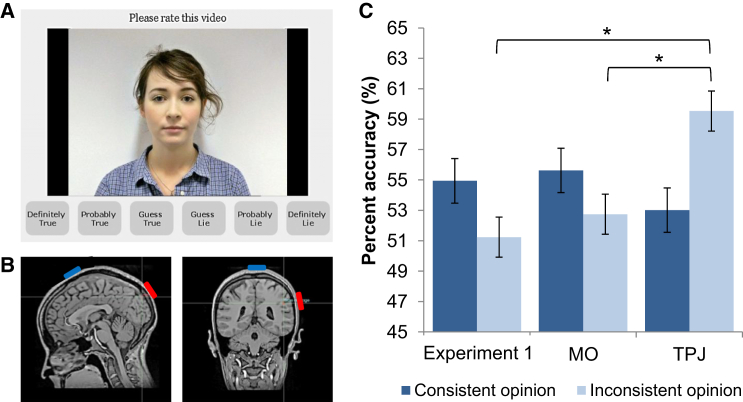
Example Lie-Detection Trial, Placement of Electrodes for rTPJ and MO Groups, and Visual Display of the Results (A) Example trial from the video-mediated lie-detection task. (B) Placement of anodal (red) and cathodal (blue) electrodes for both the MO (left) and TPJ (right) groups. (C) Percentage accuracy when the veracity of opinion statements consistent and inconsistent with the participant’s own opinion was judged. The data presented are from experiment 1 and the MO and TPJ stimulation (experiment 2) groups. ^∗^ denotes significant difference at p < 0.05, and error bars represent the SEM.

It is well-established that self-representations can interfere with representation of another even when task irrelevant. The act of planning or executing an action interferes with the perception of an incongruent action performed by another [10], one’s own affective state biases perception of another’s incongruent affective state [11], one’s own visual perspective interferes with the representation of another’s spatially inconsistent visual perspective [12], and the contents of one’s own mental states interfere with representation of those of another when they differ from our own [13]. A body of previous research has highlighted how each of these social abilities recruits a mechanism to enable the individual to control, or switch between, representation of the self and of others to avoid interference between inconsistent representations, such that representation of the self is enhanced and the other inhibited, or representation of the other is enhanced and the self inhibited according to task demands [14–18]. These results raise the possibility that holding an opinion inconsistent with that expressed by another may interfere with the ability to judge the veracity of the expressed opinion and that increasing the ability to inhibit representation of one’s own opinion and enhance that of the other may result in improved lie-detection performance when opinions are inconsistent. Accordingly, over two experiments, participants were asked to complete a video-mediated lie-detection task based on the false-opinion paradigm [19–21] (Figure 1A), in which they were asked to rate whether an individual (the “sender”) had expressed their true or a false opinion.

Experiment 1 sought to establish evidence for the hypothesized opinion inconsistency effect by comparing lie-detection performance for opinion-consistent (where the participant’s opinion matched the sender’s stated opinion) and opinion-inconsistent (where the participant’s opinion was opposite to the sender’s stated opinion) statements. A group of healthy adult volunteers (n = 63; mean age = 33.5, SD = 6.4; 44 female) were asked to complete the lie-detection task after completing a questionnaire ascertaining their views on a number of controversial topics. As hypothesized, when rating the veracity of opinion statements expressed by senders (i.e., whether the sender had presented their true opinion), participants were significantly more accurate when the view expressed by the sender was consistent with their own view (mean percent accuracy ± SEM: 54.9% ± 0.8%) than when inconsistent (51.2% ± 1.1%; *t*(62) = 3.02, p = 0.004, *d* = 0.49).

Experiment 2 tested the efficacy of anodal transcranial direct current stimulation (tDCS), a form of non-invasive electrical brain stimulation, of the temporoparietal junction (TPJ) to improve lie-detection accuracy on opinion-inconsistent trials. A series of recent studies suggest that the TPJ plays a crucial role in the mechanism that enables the control of representations of the self and of others [2–5, 15, 18]. These studies suggest the TPJ allows representation of the self to be inhibited and the other enhanced, or the self enhanced and the other inhibited, and that this process is recruited in theory of mind, imitation inhibition, and visual perspective taking [17, 18, 22–25]. In line with previous demonstrations across these different social domains, it was predicted that stimulation of the TPJ would allow representation of one’s own opinion to be inhibited and representation of the other’s opinion to be enhanced, leading to a reduction in the opinion inconsistency effect and improved lie-detection performance on opinion-inconsistent trials.

Thirty-three healthy adult participants (mean age = 24.2, SD = 4.6; 18 female) underwent 20 min of tDCS, over either the right TPJ (rTPJ) or a mid-occipital (MO) control region (Figure 1B), prior to completing the same video-mediated lie-detection task but after their own opinions were obtained. The two groups did not differ in their age, gender, or opinions on the topics discussed (p > 0.05), and there was no significant difference in overall lie-detection performance between the two groups (*t*(31) = 0.93, p = 0.361). Moreover, overall performance (collapsing across opinion-consistent and -inconsistent trials) did not differ significantly from the population derived average of 54% [1] in either the TPJ (*t*(15) = 1.18, p = 0.257) or MO (*t*(16) = 0.25, p = 0.804) group.

Lie-detection performance was then analyzed using a mixed-effect two-way ANOVA with a within-subjects factor of opinion consistency (two levels: performance on opinion-consistent versus opinion-inconsistent trials) and a between-subjects factor of stimulation group (two levels: stimulation of TPJ versus MO). As predicted, there was a significant opinion consistency × stimulation group interaction (*F*(1,31) = 7.74, p = 0.009, *η*_*P*_^*2*^ = 0.20), whereby participants who underwent rTPJ stimulation were significantly more accurate when the sender’s expressed opinion was inconsistent with their own opinion (59.5% ± 1.6%) when compared to those administered MO control stimulation (52.7% ± 1.2%; *t*(31) = 3.32, p = 0.002, *d* = 1.15). Conversely, there was no significant difference in lie-detection performance between stimulation groups during trials in which the sender and participant’s opinions were consistent (*t*(31) = 1.03, p = 0.313; Figure 1C).

The significant improvement in lie detection after tDCS of the TPJ, specific to situations in which one must suppress one’s own opinion to judge the veracity of another’s statement, supports both the involvement of a self-other control mechanism in lie detection and the involvement of the TPJ in this process. Results suggest that in situations of conflict between one’s own opinion and that of another, one must inhibit the representation of one’s own opinion and enhance that of the other in order to successfully discriminate between a truth and a lie.

The relatively modest improvement in absolute accuracy observed after TPJ stimulation is in accordance with both the size of the consistency effect observed in experiment 1 and the degree of individual differences in the population as a whole. A mean success rate of 54% across all published studies (albeit with an unknown proportion of opinion-consistent and -inconsistent trials), with a measurement-corrected SD of just 0.8% [1], means that the increase in lie detection ability after TPJ stimulation is therefore not trivial with respect to population-level statistics. Indeed, the mean percentage accuracy of the TPJ stimulation group on opinion-inconsistent trials was significantly greater than the population-derived figure of 54% (*t*(15) = 3.33, p = 0.005, *d* = 1.17) but on consistent trials was not (*t*(15) = 0.46, p = 0.651). Accuracy in the MO stimulation group did not differ significantly from 54% on either consistent or inconsistent trials (p > 0.05).

These data are promising for understanding the mechanisms involved in, and factors affecting, lie-detection performance. However, care should be taken when considering the use of tDCS to improve lie detection in real-world situations, where even the 87% lie-detection accuracy achieved by polygraphs [26] does not ensure that truths are not flagged as lies and vice versa—especially in situations where the base rate of lying is likely to be low, such as in employment screening. Also, although the time course of the improvement in lie detection was not measured in this study, it is likely to be short lived: with the stimulation parameters used in the current experiment, one would only expect effects to last for approximately 90 min [27]. Thus, it seems that much of the value of these results lies in their theoretical implications for the processes involved in lie detection, for the applicability of self-other control mechanisms to higher-level social cognition, and for our knowledge of TPJ function.

The specificity of the improvement in lie detection (on opinion-inconsistent trials only) suggests that stimulation does not act on a general process involved in social cognition, such as theory of mind [28] or emotion recognition [29]. Rather, the results are best explained in the context of the mechanism of self-other control. Self-other control has been shown to underlie the role of the TPJ in various social functions on the basis of organic lesions [25], experimentally induced disruption [30], and facilitation [3–5] of TPJ function and through the use of neuroimaging methods such as fMRI [14].

With regard to the full mechanism by which opinion inconsistency produces impaired lie-detection performance, it is plausible that individuals who hold strong opinions for or against a topic have been exposed to similar arguments in support of their position and against the opposite view [31], allowing them to recognize the match between a position and the most common justifications for that position when a sender has an opinion consistent with their own. If individuals are presented with an opinion counter to their own, their lack of experience relating to justifications supporting that stance on the topic means they may be ill equipped to judge the appropriateness of the justification and therefore the veracity of the stated opinion. It is important to realize, however, that the effect of TPJ stimulation is unlikely to be attributable wholly to inhibition of one’s own opinion: if inhibition were the only effect of stimulation, then performance on inconsistent trials would have been equivalent, rather than superior, to performance on consistent trials. Instead, results suggest that representation of the other was also enhanced, in line with previous findings on self-other control and TPJ stimulation [3–5] and with theories attributing an attentional, switching, or gating role to the TPJ [16, 32, 33]. A necessary subsequent focus for research is to identify the mechanism by which an enhanced representation of the other results in an increased ability to detect lies.

In conclusion, experiment 1 established self-other interference effects in the context of lie detection, where representations refer to the opinions of the self and the other. Participants were less accurate at distinguishing truthful from false opinions when the sender’s opinion was inconsistent with their own. Experiment 2 demonstrated that this performance interference effect could be reduced through anodal stimulation of TPJ, improving lie detection specifically on those trials in which this effect was most prominent. These results suggest that boosting the ability to control representations of the self and other—in this case inhibiting one’s own opinion in order to more accurately represent that of the other—can improve lie detection in opinion-inconsistent situations.

## Experimental Procedures

### Lie-Detection Task

In both experiments 1 and 2, participants began by completing an “opinion questionnaire” in which they gave their opinion on 20 topics. For each item they rated the degree to which they agreed or disagreed with the topic on a six-point scale (with an answer of “1” demonstrating strong agreement, and “6” strong disagreement, with a topic). Example items on the questionnaire include “euthanasia,” “medical abortion,” “genetically modified foodstuffs,” and “animal testing.” During the lie-detection task, participants watched a series of 80 randomly ordered video clips of individuals (“senders”) expressing their views, as well as a brief justification of their view, on the same topics included in the opinion questionnaire. These took the form of “I am in favor of euthanasia because everyone deserves a chance to die with dignity.” After watching each video, participants were asked to rate whether the sender had presented their true opinion or whether they had lied, on a 6-point scale (1, definitely true; 6, definitely lie; see Figure 1A). The task took a total of 25 min.

The stimulus set comprised the same set of 40 truthful and 40 deceptive statements for all participants, conveyed by 20 different individuals (ten males and ten females). The stimulus set contained four video statements about each of the 20 topics contained in the opinion questionnaire—two truths and two lies—and in a fully factorial design, two statements were spoken in agreement and two in disagreement with each topic. The videos were recorded during a previous experiment and were all provided by individuals who had strong opinions for or against each topic (ratings of “1” or “6” on the opinion questionnaire). Note that the factorial combination of truthful and deceptive statements, for and against each topic, means that the observed improvement in lie detection performance on opinion-inconsistent trials after stimulation is an effect on accuracy rather than bias in both experiment 1 and experiment 2.

### Data Analysis

Trials were divided into opinion-consistent and opinion-inconsistent trials on a trial-by-trial basis according to the sender’s expressed opinion and the participant’s opinion as reported on the opinion questionnaire. For example, if a participant listed their opinion as “against” a topic, a trial in which the sender expressed their opinion as “against” the topic was classed as an opinion-*consistent* trial, whereas a trial in which the sender expressed their opinion as “for” the topic was classed as an opinion-*inconsistent* trial. Participants’ responses on each trial were dichotomized as either a “truth” (reponses 1–3) or a “lie” (responses 4–6) judgment to account for individual differences in the use of the extremities of the rating scale. The percentage accuracy of judgments constituted the measure of lie detection performance, which was compared in experiment 1 for opinion-consistent and -inconsistent trials using a paired-sample t test. In experiment 2, lie-detection performance was analyzed using a mixed-effect two-way ANOVA (with opinion consistency as the within-subjects factor and stimulation group as the between-subjects factor). Two participants were excluded prior to data analysis in experiment 2: one from the TPJ stimulation group, who responded “true” to over 90% of trials, and one from the MO stimulation group, who completed less than 20% of trials, leaving too few trials for analysis.

It should be noted that the design of both experiment 1 and experiment 2 followed current best practice guidelines by comparing lie detection performance on the critical condition of interest (opinion-inconsistent trials) with a within-participant baseline (opinion-consistent trials), which also served as an extremely closely matched control condition. Indeed, participants were performing the same task, to videotaped statements from the same people, concerning the same topics, on both opinion-consistent and opinion-inconsistent trials.

### Experiment 2 tDCS Protocol

Participants were randomly assigned to one of two tDCS groups: rTPJ (n = 17) or MO control (n = 18). All participants were healthy volunteers, with no known developmental or neurological disorders, normal or corrected-to-normal vision, and no contraindications to tDCS. Prior to study completion, all participants were naive to the aims of the experiment.

All participants underwent anodal stimulation, induced with two saline-soaked surface sponge electrodes (35 cm^2^) and delivered by a battery-driven, constant current stimulator. According to group assignment, the anodal electrode was placed over central parietal 6 (CP6) for rTPJ stimulation and occipital zero (OZ) for MO control stimulation (electroencephalography 10/20 system). MO was used as an active control site as there was no a priori reason to assume that stimulation to this region would differ from baseline. The cathodal electrode was placed over the vertex as a reference point, individually measured on each participant (Figure 1B). A weak electrical current (1 mA) was delivered offline (preceding the task) for a total of 20 min, following the procedure used by Santiesteban and colleagues [4, 5], as these effects are reported to be more robust than online stimulation. The effects of stimulation with these parameters have been demonstrated to last for 90 min after stimulation [27]. Participants completed the opinion questionnaire prior to stimulation, whereas the lie detection task was completed within the critical 90 min window after stimulation. Both experiments 1 and 2 received full ethical approval by the local Research Ethics Committees (Birkbeck, University of London and Institute of Psychiatry, Psychology, and Neuroscience, King’s College London, respectively).
